# Insights into the structural dynamics and helicase-catalyzed unfolding of plant RNA G-quadruplexes

**DOI:** 10.1016/j.jbc.2022.102165

**Published:** 2022-06-20

**Authors:** Liu Wang, Ya-Peng Xu, Di Bai, Song-Wang Shan, Jie Xie, Yan Li, Wen-Qiang Wu

**Affiliations:** State Key Laboratory of Crop Stress Adaptation and Improvement, Academy for Advanced Interdisciplinary Studies, School of Life Sciences, Henan University, Kaifeng, China

**Keywords:** G-quadruplex, RNA helicase, RNA-binding protein, molecular dynamics, single-molecule biophysics, dG4, DNA G-quadruplex, MST, microscale thermophoresis, rG4, RNA G-quadruplex, RSM, RHAU specific motif, smFRET, single-molecule FRET

## Abstract

RNA G-quadruplexes (rG4s) are noncanonical RNA secondary structures formed by guanine (G)-rich sequences. These complexes play important regulatory roles in both animals and plants through their structural dynamics and are closely related to human diseases and plant growth, development, and adaption. Thus, studying the structural dynamics of rG4s is fundamentally important; however, their folding pathways and their unfolding by specialized helicases are not well understood. In addition, no plant rG4-specialized helicases have been identified. Here, using single-molecule FRET, we experimentally elucidated for the first time the folding pathway and intermediates, including a G-hairpin and G-triplex. In addition, using proteomics screening and microscale thermophoresis, we identified and validated five rG4-specialized helicases in *Arabidopsis thaliana*. Furthermore, DExH1, the ortholog of the famous human rG4 helicase RHAU/DHX36, stood out for its robust rG4 unwinding ability. Taken together, these results shed light on the structural dynamics of plant rG4s.

G-rich DNA/RNA can form four-strand noncanonical G-quadruplex (G4) structures, which comprise Hoogsteen-bonded planar G-quartets and be further stabilized by monovalent cations such as K^+^ or Na^+^ (1). They are highly stable and implicated in many crucial cellular processes (1, 2, 3). Their folding dynamics are closely related to human health (4, 5) and can regulate plant growth, development, and adaption (6, 7, 8). Although DNA G4s (dG4s) have been studied well from different perspectives, RNA G4s (rG4s) have only gradually received attention in recent years (1, 9). Compared to dG4s, because the vast majority of RNA exists in the cell as a single strand without the restriction of complementary strands, G-rich RNA is easier to fold into more thermodynamically stable rG4 structures (10, 11). In addition, although dG4s can fold into different kinds of topological structures, because of the presence of 2′-OH, rG4s predominantly fold into parallel structures (11, 12).

rG4s exist in mRNA and noncoding RNA (6, 9, 13, 14) and can fine-tune both DNA and RNA metabolism (3). Currently, research on rG4s is mainly focused on animals. As multicellular organisms, plants have evolved independently and provide oxygen and food for animals. As early as 2010, Mullen *et al*. predicted that rG4s might play important roles in *Arabidopsis* (15). Furthermore, in 2012, the folding thermodynamics of plant rG4s were also systematically studied by the same group (16). In 2015, Kwok *et al*. (7) were the first to show *in vivo* that an rG4 structure in the 5′ UTR of *Arabidopsis* mRNA could regulate the ATR protein expression by blocking its translation. In 2018, Cho *et al*. (8) showed that an rG4 in the SMXL4/5 5′ UTR could control phloem differentiation. In 2019, Zhang *et al*. (17) discovered that an rG4 in SHR mRNA could trigger RNA phase liquid–liquid phase separation. In 2020, Yang *et al*. reported that rG4s were widely present in *Arabidopsis* and rice, and they played an important role in modulating plant growth as demonstrated by rG4 sequencing and phenotypic experiments (6).

The structures of rG4s are highly dynamic in cells (18, 19), and the structural dynamics regulate related cell activities (3). Thus, it is critical to understand the structural dynamics of rG4s to understand their cellular and molecular functions. Although there are a few studies on rG4 folding-unfolding intermediaries and dynamics, using both bulk (16, 20) and single-molecule methods (21, 22), the underlying mechanism have not been well revealed. Studying this process is more meaningful for plants because the exterior environment can influence ion concentrations and temperature in plant cells, which can modulate the folding dynamics of rG4 structures (16). In addition, the dynamics of rG4s can be modulated by rG4-binding proteins (3, 9), in which helicases are confirmed to play a key role in rG4 biology *via* resolving these highly stable structures. Therefore, the identification of rG4-specialized helicases is quite important when explaining the functions of rG4s. In humans (4, 9) and yeast (23, 24), many helicases have been reported to unfold rG4 structures. Although rG4s are also important in plants (6), no specialized helicases have been reported to interact with rG4s in plants. This knowledge gap limits the study of rG4 function in plants.

In this research, four plant rG4s were chosen and their folding was confirmed by CD. Afterward, different concentrations of KCl were used to induce their folding, and four folding states, including two folding intermediates (G-hairpin and G-triplex), were captured by single-molecule FRET (smFRET) and verified through substrate mutation. In addition, proteomics screening identified five rG4-specialized helicases from *Arabidopsis*, and their specialization was confirmed by the microscale thermophoresis (MST) method. Further, the unwinding abilities of these five helicases were investigated. These findings may improve the understanding of rG4 functions in plants.

## Results

### The folding of four selected rG4 sequences depends on the concentrations of KCl

**Figure 1:**
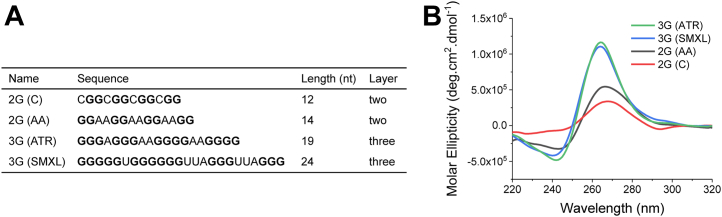
**Four selected rG4 forming sequences can fold into parallel G4 structures.***A*, sequence information of four selected rG4 sequences. *B*, CD spectra of rG4 in 25 mM Tris–HCl, pH 8.0, and 100 mM KCl, showing an ∼265 nm peak. rG4, RNA G-quadruplex.

To investigate the properties of rG4s, four reported plant rG4-forming sequences were selected from the literature (Fig. 1*A*), including two two-layer rG4s named 2G (C) and 2G (AA) (6, 16) and two three-layer rG4s referred to as 3G (ATR) and 3G (SMXL) (7, 8). Using the CD spectra, it was further confirmed that they could properly fold into parallel G4 structures in 100 mM KCl (Fig. 1*B*), showing an approximately 265 nm peak, consistent with previous reports (11, 12).

**Figure 2:**
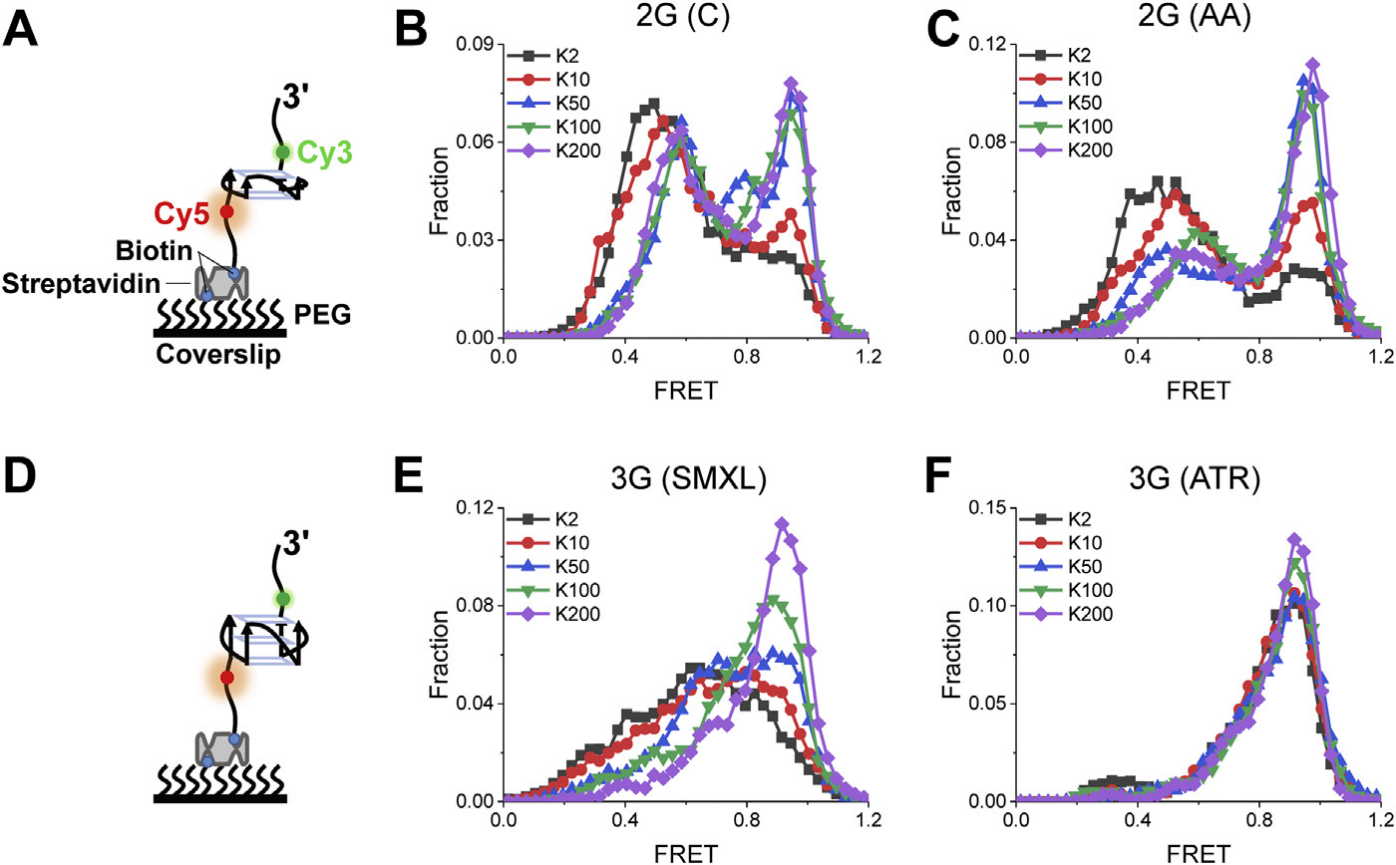
**smFRET distributions of 2G (C), 2G (AA), 3G (ATR), and 3G (SMXL) for different concentrations of K**^**+**^**.***A* and *D*, schematic diagrams of two-layer and three-layer rG4 structures. *B*, *C*, *E*, and *F*, FRET distributions of two-layer rG4 2G (C) and 2G (AA) and three-layer rG4 3G (SMXL) and 3G (ATR) under different concentrations of K^+^. Kn is used to represent reaction buffer in the text, where n indicates the concentration of KCl; for example, K100 represents 25 mM Tris–HCl at pH 8.0 and 100 mM KCl. rG4s, RNA G-quadruplexes; smFRET, single-molecule FRET.

rG4 structures are formed in ssRNA in cells. To mimic this structural environment and monitor the folding dynamics of rG4s at the single-molecule level, the fluorescently labeled substrates of these four rG4s were designed (Fig. 2, *A* and *D*). Each substrate was constructed with an ssRNA containing Cy3 and Cy5 to the ss-rG4 junctions and modified by biotin at the 5′ end for immobilization to the PEG-modified surface of a coverslip with streptavidin. Thus, FRET signal change could sensitively report the structural change of rG4s. Based on the principle of FRET, folded rG4 should correspond to the highest FRET value, while unfolded ssRNA should correspond to the lowest FRET value. The formation of rG4 structures is induced and stabilized by K^+^ and Na^+^ (1). In plant cells, the concentration of K^+^ is two orders of magnitude higher than that of Na^+^ (25, 26); therefore, K^+^-induced structures are biologically more relevant. In addition, the concentration of K^+^ is physically charged in response to cellular stress. With the increase of KCl concentration, the FRET efficiency gradually shifted toward high FRET values (Fig. 2), indicating that K^+^ induced the formation of rG4 structures as expected (16). Compared to the three-layer rG4s, the two-layer rG4s did not appear to change much. This difference may be due to their thermal stability. Compared to the two-layer rG4s, the three-layer rG4s are always stable. This observation is consistent with a published result that the folding of two-layer rG4s need higher K^+^ concentration (16), which can reach up to ∼600 mM in plant cells under drought stress (16, 27).

### rG4s show four folding states

**Figure 3:**
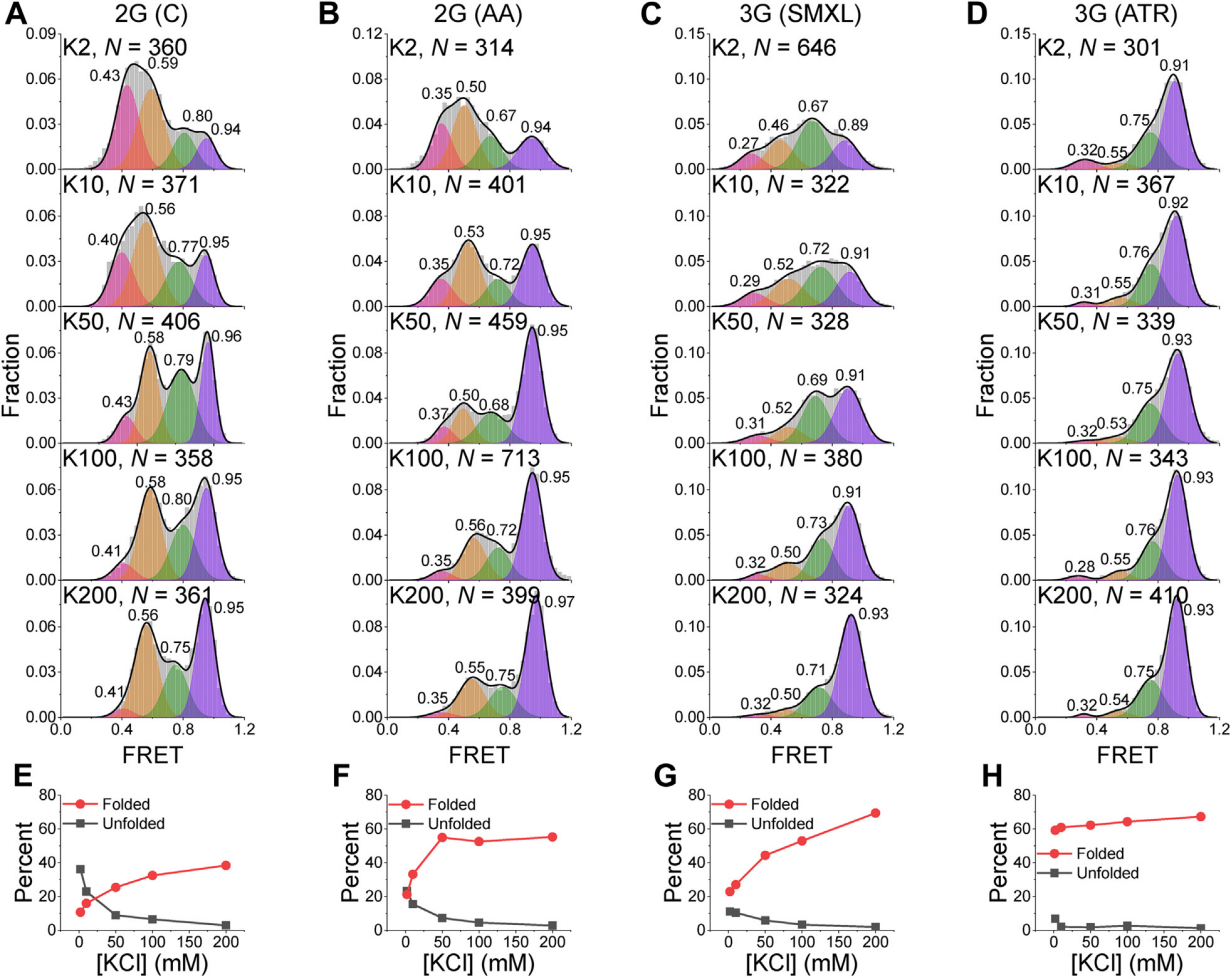
**rG4 structures show four folding states.***A*–*D*, histogram fitting of four rG4 structures using multipeak Gaussian distributions shows four folding states for all substrates, and the FRET value of each state is marked. *E*–*H*, the well-folded and unfolded fractions of corresponding RNA substrates with the change in K^+^ concentration by calculating the ratio of peak areas. *N* represents the number of traces used for analysis. rG4, RNA G-quadruplex.

The K^+^-induced rG4 folding provided an opportunity to study the folding intermediates and pathways of rG4s. To dissect the intermediates and dynamics of rG4s, the FRET histograms were fitted to recognize the folding states. It was found that they were well fitted by four peaks using multipeak Gaussian distributions (Fig. 3, *A*–*D*). Therefore, the representative traces were also identified as four states by hidden Markov modeling, and the transition density plots of all four rG4s were built (Fig. S1), which further confirmed the multipeak fitting. Based on the principle of FRET, it was clear that the lowest FRET state should correspond to completely unfolded sequences (ssRNA) and the highest FRET state should parallel G4 (Fig. 1*B*). This was consistent with the fact that, with increasing KCl concentrations, the proportion of the lowest FRET value gradually decreased; accordingly, the proportion of the highest FRET value gradually increased (16) (Fig. 3, *E*–*H*). It also made sense that the FRET values of the unfolding states gradually decreased with the length increase of single strands (Fig. S2*A*), and the FRET values of folding states decreased with the increasing number of tetrad layers (Fig. S2*B*).

**Figure 4:**
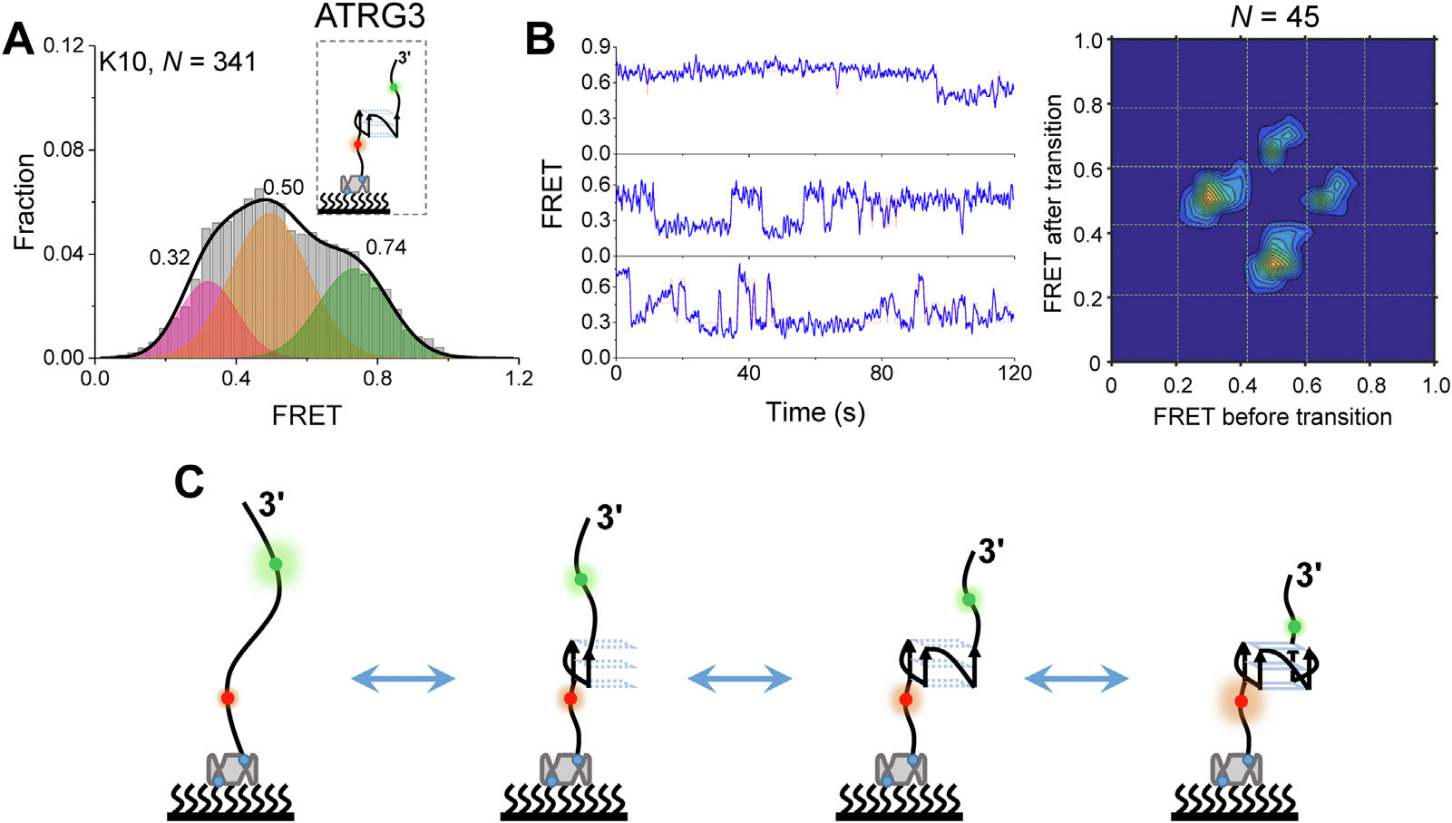
**Proposed folding-unfolding pathway of rG4 structures.***A*, schematic view and FRET histogram of ATRG3 in 25 mM Tris–HCl, pH 8.0, and 10 mM KCl, showing three states. *B*, representative FRET traces of ATRG3 (*left panel*). Dynamic states are determined by hidden Markov modeling (*red line*), and the transition density plots are built, showing the transitions between these three states. *C*, proposed folding-unfolding pathway of rG4s, which contains two intermediate states of G-hairpin and G-triple. *N* represents the number of traces used for analysis. rG4, RNA G-quadruplex.

Because the four rG4s were parallel structures (Fig. 1*B*), the remaining two states should not be other G4 topologies and thus should be the folding intermediate states of rG4s. For dG4s, G-hairpin and G-triplex have been well identified as folding intermediates (28, 29, 30, 31, 32, 33). In terms of rG4s, G-hairpin and G-triplex RNA were also proposed as folding intermediates of rG4s (20, 34), even though they were not directly captured experimentally in real time. Therefore, it was easy to speculate that the intermediates were very likely to be RNA G-hairpin and G-triplex. If so, the existence of G-hairpin and G-triplex should be independent of rG4. To confirm this speculation, the last column of 3G (ATR) (Fig. 4*A* insert, referred to as ATRG3) was mutated to detect the formation of G-hairpin and G-triplex. As expected, three Gaussian peaks at 0.32, 0.50, and 0.74 were captured (Fig. 4*A*), consistent with the representative traces (Fig. 4*B*). ATRG3 can only fold into G-hairpin and G-triplex; therefore, the peaks 0.50 and 0.74 should be G-hairpin and G-triplex, respectively. The FRET values of ATRG3 in Figure 4*A* were close to the lower values of 3G (ATR) in Figure 3*D*, strongly supporting the intermediate states were G-hairpin and G-triplex, and the missing FRET of ∼0.92 was further confirmed to consist of well-folded rG4 structures. Thus, the folding-unfolding pathway of rG4s (Fig. 4*C*) containing G-hairpin and G-triplex as intermediates was proposed. A previous study reported that rG4 unfolding by DHX36 showed four states. However, the states were not assigned to specific structures (35). To the best of our knowledge, this study represents the first time that both G-hairpin and G-triplex have been directly captured experimentally during rG4 folding-unfolding processes in real time.

In early studies, at physiological K^+^ concentrations of ∼140 mM *in vitro*, three-layer rG4s were reported to be well folded (16, 19), and even the relatively unstable three-layer dG4s were also able to fold well (36). However, in the present single-molecule experiments, a considerable proportion was not fully folded (Fig. 3), which may have been averaged in bulk assays. To resolve this inconsistency, the present study designed a DNA substrate named 3G (ATR)-DNA by replacing the RNA sequence of 3G (ATR) with DNA to verify the reliability of smFRET. CD spectral analysis showed that 3G (ATR)-DNA folded into parallel G4 structures, and it was confirmed that it was less stable than RNA 3G (ATR) through melting (Fig. S3). Under the same experimental conditions, it was found that 3G (ATR)-DNA also showed four Gaussian peaks (Fig. S4, *A*–*C*), and the peak values were close to those of RNA 3G (ATR) (Fig. 3*D*). G-hairpin and G-triplex have been well demonstrated to be the intermediates of dG4s (28, 29, 30, 31, 32, 33), which further supports the rG4 folding-unfolding proposal in the present study (Fig. 4*C*). In addition, the unfolded fraction of DNA was higher than that of RNA and the completely folded fraction was less than that of RNA (Fig. S4*D*); this was consistent with the stability results (Fig. S3). Therefore, it was reasoned that this difference may have been caused by the flanking sequences connected to rG4s. Recently, it was found by us that the proximal ssRNA impaired the stabilities of rG4 structures *in vitro* (22). In addition, rG4 functions were also reported to be context dependent *in vivo* (37). This fact can also explains why some proteins are needed to promote rG4 folding (3, 8).

### Identification and confirmation of plant rG4-binding helicases

**Figure 5:**
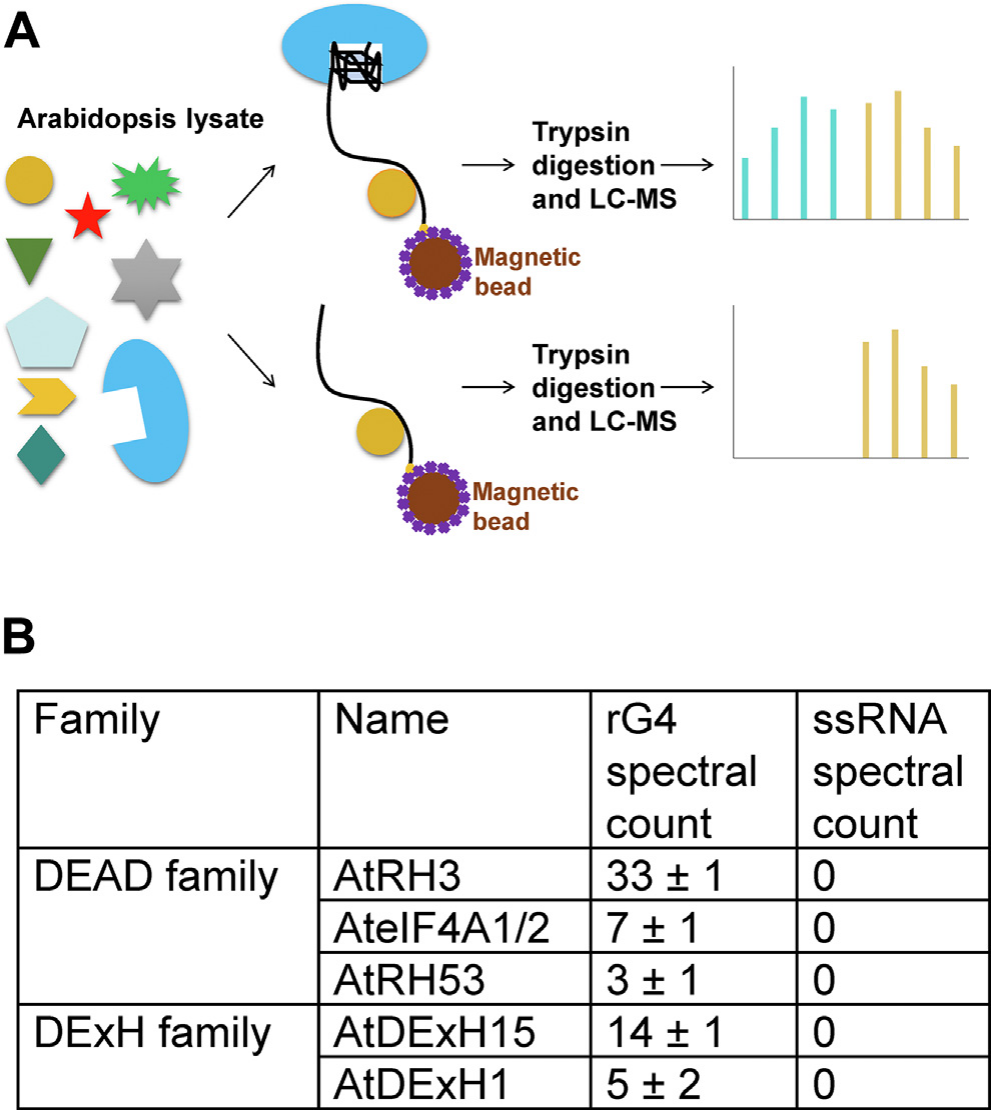
**Identification of rG4 helicases in *Arabidopsis*.***A*, scheme of proteomics screening. rG4-binding proteins are selected through binding the rG4 bait, and ssRNA is used to subtract nonspecific interactions. Spectral counting is used to determine the abundance of proteins. *B*, five rG4-specialized helicases are identified in rG4 proteomics screening. rG4, RNA G-quadruplex.

As described previously, the dynamics of rG4s induced by K^+^ were studied, the folding intermediates were revealed, and the folding pathway was proposed. In cells, the structural dynamics can also be modulated by rG4-binding proteins, in which helicases exhibit strong rG4 unwinding activity (38). In animals, many rG4-specialized helicases have been reported (4, 10) and are indispensable for explaining the functions of rG4s. However, surprisingly, no plant rG4 helicases have been reported until now. To identify plant rG4 helicases, rG4 proteomics screening was performed (Fig. 5*A*), in which rG4 served as bait to pull down rG4-binding proteins and ssRNA was used for comparison, followed by SDS-PAGE and mass spectrometric analysis. The comparison of spectral counts was used to determine protein abundance (24, 39). Five rG4 helicase candidates were identified using this method (Fig. 5*B*). Three of them belonged to the DEAD helicase family, and two of them were DExH family helicases. The mass spectrometric spectra of representative peptides are shown in Fig. S5. It should be noted that the eIF4A ortholog in human cells has been reported to be a specialized rG4 helicase (40), implying the reliability of the pull-down results in the present study.

**Figure 6:**
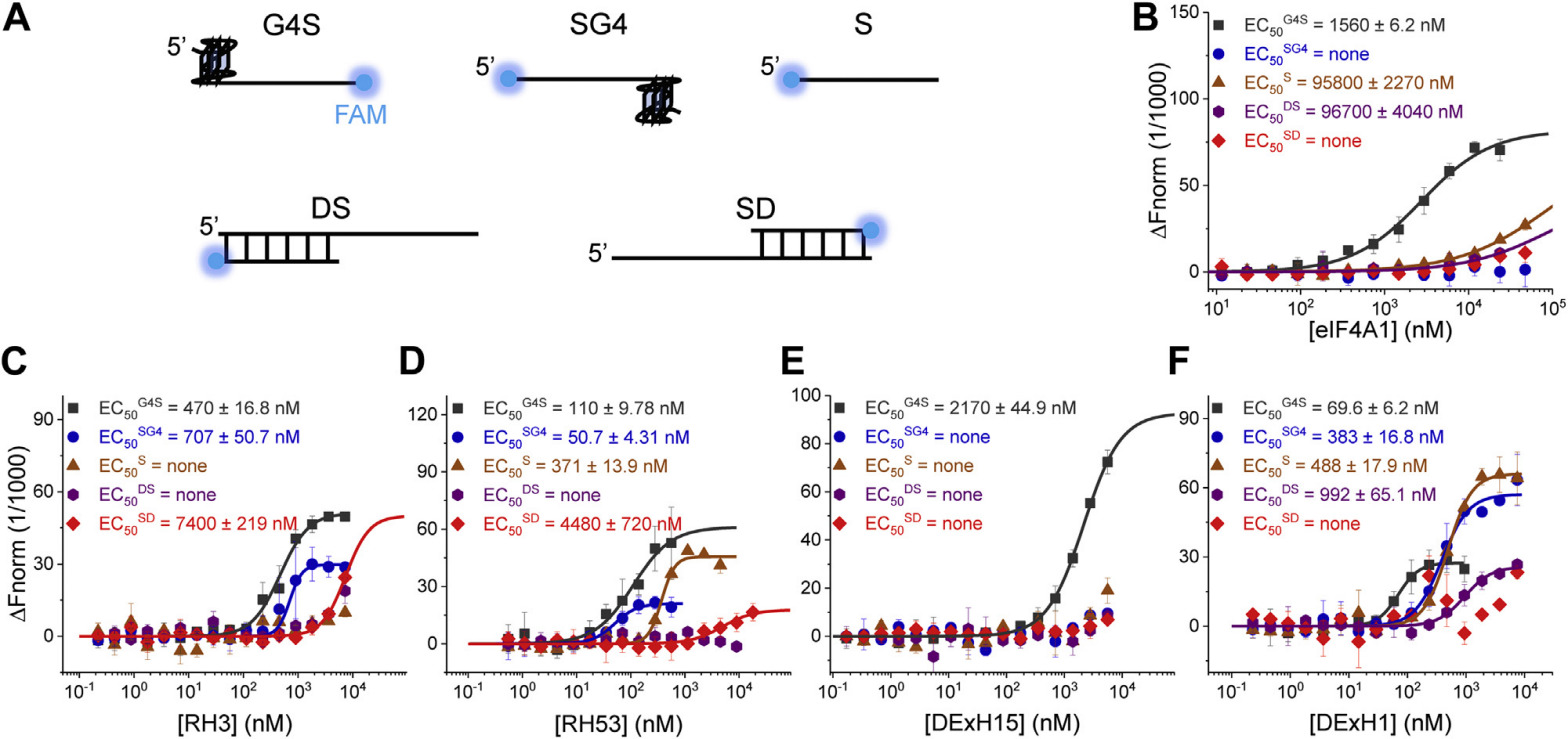
**Binding affinity measurements of five helicases using MST.***A*, schematic diagram of five checked substrates. *B*–*F*, binding information of eIF4A, RH3, RH53, DExH1, and DExH15 in five substrates. The concentration of each fluorescein-labeled substrate is fixed at 20 nM, and the values of normalized fluorescence (Fnorm) are detected as the protein concentration increased. The EC_50_ values indicate that rG4-containing substrates are their optimal substrates because smaller EC_50_ values represent greater binding affinity. MST, microscale thermophoresis; rG4, RNA G-quadruplex.

To further confirm that these five helicases were rG4 specialized, they were expressed and purified to homogeneity (Fig. S6), and their binding affinity to different kinds of RNA substrates was checked using MST (Fig. 6*A*). The comparable binding affinity supported their specialization in rG4 recognition (Fig. 6, *B*–*F*) because smaller EC_50_ values represent greater binding affinity (29). RH3 and RH53 were SUMO-tagged in the MST experiments. To exclude the influence of the SUMO tag, its binding with these substrates was measured and no obvious interaction was detected (Fig. S7).

### DExH1 and DExH15 can unfold rG4 structures in the 3′-5′ direction

**Figure 7:**
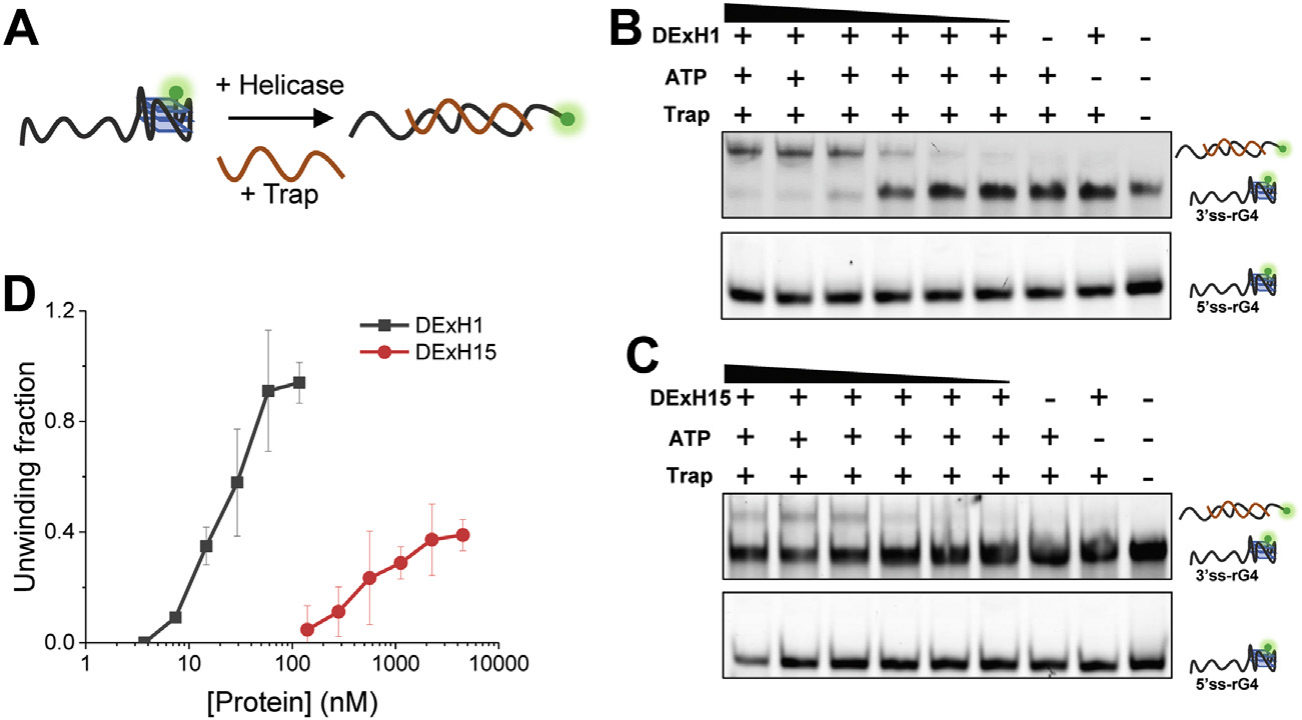
**Helicase assays of DExH1 and DExH15.***A*, scheme of experimental strategy. Only when the rG4 structure is unfolding, can the unlabeled ssRNA trap pair with the rG4-containing strand to form the double strand. *B* and *C*, gel shift of DExH1 (*B*) and DExH15 (*C*) unwinding activity on 3’ss-rG4 and 5’ss-rG4. The concentration of each fluorescein-labeled substrate is fixed at 160 nM. The concentration of DExH1 is from 117 nM to 3.7 nM and the concentration of DExH15 is from 4500 nM to 140 nM. For controlled experiments, the concentration of DExH1 is 117 nM and the concentration of DExH15 is 4500 nM. In the absence of helicases or ATP, there is no detectable unwinding despite the presence of traps; therefore, their unwinding is ATP-dependent and should not result from helicase binding with the assistance of traps. *D*, quantitative analyses of DExH1 and DExH15 unwinding of 3'ss-rG4, showing DExH1 is much more active than DExH15 under the same experimental conditions. rG4, RNA G-quadruplex.

After identifying and confirming rG4-specialized helicases, this study sought to determine whether they had rG4 unwinding ability. Gel shift has been reported to be an intuitive and reliable method to study rG4 unwinding (41). Considering this, the Cy3-labeled 3′ tail rG4 and 5′ tail rG4 structures were examined, and it was reported that they were unable to base-pair by unlabeled ssRNA traps unless they were unfolded by helicases (Fig. 7*A*). The five helicases were used to unwind both 3’ss-rG4 and 5’ss-rG4. It was found that only DExH1 and DExH15 showed obvious rG4 unwinding activity (Fig. 7, *B* and *C*). None of the three DEAD helicases showed rG4 unwinding under the experimental conditions, even when their concentration reached as high as the micromolar levels (not shown). The gel shift results clearly showed that DExH1 and DExH15 could resolve 3′ tail rG4 (Fig. 7, *B* and *C*, upper panels), but they could not unfold 5′ tail rG4 under the same experimental conditions (Fig. 7, *B* and *C*, bottom panels), implying that they were 3′-5′ helicases. This was consistent with their preferential binding of 3′ tail rG4 in the MST experiment (Fig. 6, *E* and *F*). As control experiments, no unwinding occurred in the absence of DExH1/DExH15 or ATP (Fig. 7, *B* and *C*, upper panel), implying that the rG4 unwinding of these two helicases is ATP dependent, and the unwinding should not result from helicase binding with the assistance of traps. Comparison made it clear that DExH1 was much more active than DExH15 in terms of rG4 unwinding (Fig. 7*D*).

RNA helicases can be classed into DEAD and DExH helicases, which share different unwinding mechanisms (42). The three DEAD helicases did not show rG4 unwinding ability, which may be explained from two aspects. First, some of them may need assistant proteins to unwind G4 structures together; for example, eIF4A unwinding activity can be activated by its cofactors (43). In agreement with this, eIF4H (44) and eIF4G (45) were recently reported to selectively bind rG4s. Second, some may function in cells through G4 recognition but not unfolding, like XPB (46). DExH helicases DExH1 and DExH15 are 3′-5′ helicases, which is consistent with rG4 unwinding helicases DHX9 and DHX36 from humans. In addition, through homolog research using the BLAST tool against the human protein database, it was found that DExH1 was an ortholog of DHX36, sharing the highest identity (Fig. S8). DHX36 is one of the most well-studied rG4 helicases, and it has been proposed that DHX36 can resolve most rG4 in human cells (47). This further supported the results of the present study.

### rG4 was unfolded by DExH1 with intermediate states of G-triplex and G-hairpin

**Figure 8:**
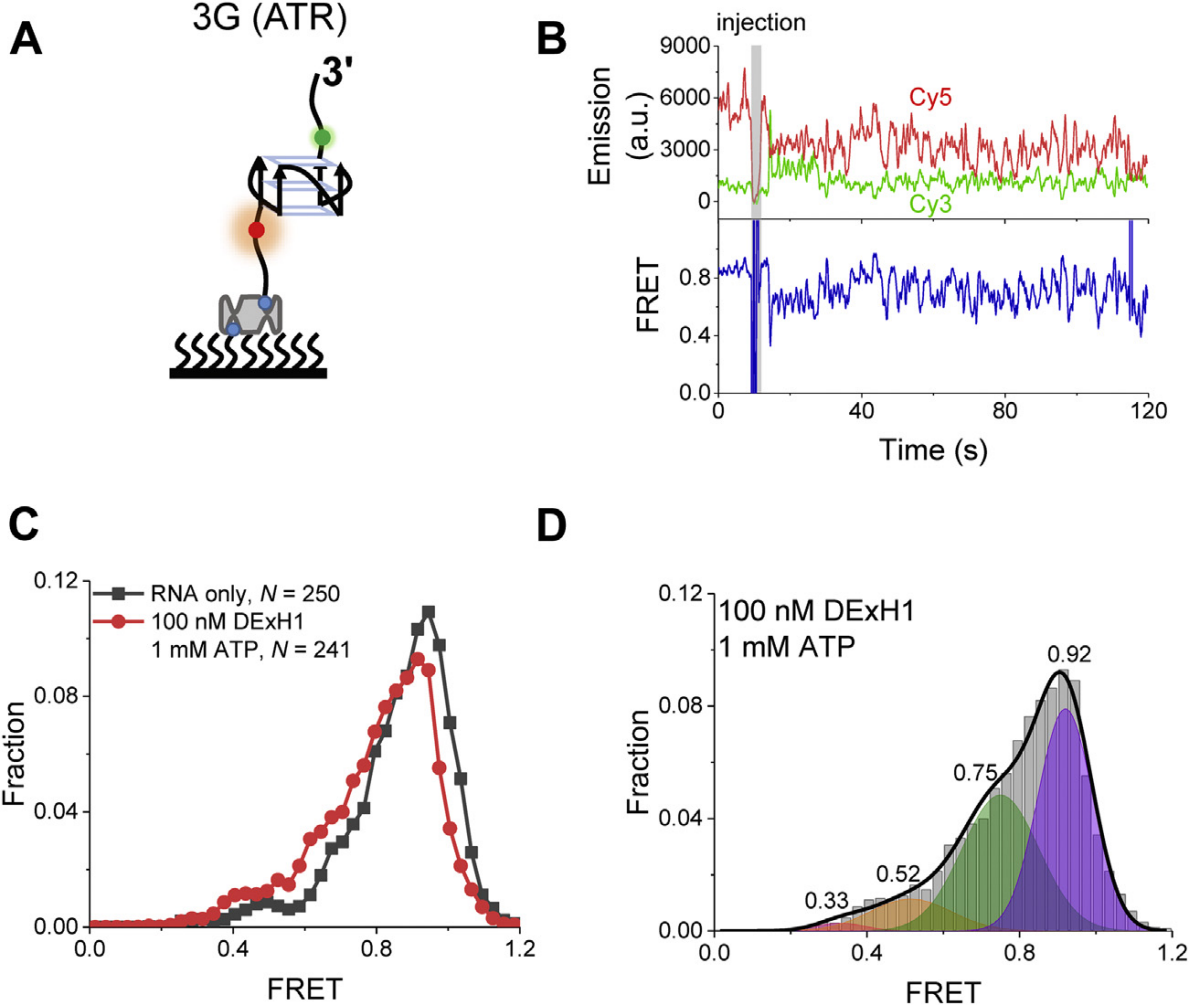
**DExH1 unwinding 3G (ATR) assay using smFRET.***A*, schematic diagram of 3G (ATR). *B*, representative time traces of fluorescence intensities of Cy3 and Cy5 (*upper panel*) after injecting 100 nM DExH1 and 1 mM ATP, and the corresponding FRET trace (*lower panel*). *C*, FRET histograms for 3G (ATR) alone, and in the presence of 100 nM DExH1 and 1 mM ATP, after 2 min of incubation. *D*, FRET distributions of 3G (ATR) in the presence of 100 nM DExH1 and 1 mM ATP. Multiple-peak Gaussian distributions are used to fit these histograms. *N* represents the number of traces used for analysis. smFRET, single-molecule FRET.

After checking the unwinding activity of identified rG4 helicases, the present study investigated the intermediate states of rG4s during helicase unfolding. Because DExH1 was more active than DExH15, DExH1 was selected to resolve 3G (ATR) (Fig. 8*A*), which was the most stable structure among four selected rG4s (Fig. 3). First, since DExH1 unwinding is ATP dependent (Fig. 7*B*) and Mg^2+^ is indispensable for the hydrolysis of ATP by helicases, it was verified that in the presence of 100 mM KCl and 5 mM MgCl_2_, there was no significant FRET change (Fig. S9). After injecting 100 nM DExH1 and 1 mM ATP into the reaction chamber, FRET fluctuations were captured (Fig. 8*B*). In agreement with this finding, after 2 min of incubation, the high FRET population decreased, accompanied by an increase of the low FRET population (Fig. 8*C*), indicating that the rG4 structures were unwound by DExH1. The distribution could also be well fitted by multipeak Gaussian distributions (Fig. 8*D*), and the peaks were close to those of dynamic folding (Fig. 3*D*), indicating that G-triplex and G-hairpin were also intermediate states during DExH1 unwinding (Fig. 4*C*). This phenomenon is consistent with the translocation-based mechanism of RHAU/DHX36 (35, 48, 49, 50). Accordingly, the fraction of folded states significantly decreased after the injection of 100 nM DExH1 and 1 mM ATP (Fig. S9*B*).

## Discussion

In recent years, the characteristics of dG4s have been reported in great detail using different methods. However, compared with dG4s, rG4s have been less well studied, especially in plants. As described previously, the present study pioneered research of the folding pathways and helicase-catalyzed unfolding of plant rG4s using single-molecule methods. These results may help elucidate their biological functions.

Under single-molecule conditions, the three-layer rG4 structures are not very well folded under physiological K^+^ conditions (Fig. 3), as previously reported (11, 16, 19). First, the small number of unfolded structures may be averaged out in bulk assays. Second, the difference may result from the ssRNA linked at both ends of rG4s, as described in a recent report (22). The substrates in the present research are more biologically meaningful because they are in line with the structural environments inside cells. In accordance with this, in both animal (3) and plant (8) cells, some factors function by directly stabilizing the rG4 structures. Therefore, in addition to the unwinding mechanisms, the molecular mechanisms of rG4 protein stabilization are also worthy of further study.

Four folding-unfolding states of rG4s were detected in both K^+^-induced dynamics (Fig. 3) and helicase unwinding (Fig. 8), and the intermediate states were assigned to G-triplex and G-hairpin. Thus, the folding pathways of both dG4s (32, 51) and rG4s are related to G-triplex and G-hairpin. The difference is that the folding of dG4s may include a variety of other G4 topologies (28, 32), while there have been no reports of rG4s with mixed structures. Recently, RHAU/DHX36 was reported to unfold rG4-containing substrate showing four states using smFRET (35). However, in these experiments, the two fluorescent dyes were not labeled at both ends of rG4. One of the dyes was labeled at the end of the ssRNA tail, so the interference of the ssRNA could not be ruled out. In addition, the study did not allocate the four states to specific molecular structures.

In the present research, five plant rG4-specialized helicases were identified for the first time using proteomics screening and MST (Figs. 5 and 6). Functional prediction indicates that they may be involved in pre-mRNA splicing and translation regulation. This information will be helpful for the study of rG4 in plants. The homologous proteins of eIF4A and DExH1 in animals have been reported to function through rG4 in animals, thus demonstrating the reliability of the present experiments. It should be noted that RHAU/DHX36, the homolog of DExH1, is the most functional rG4 helicase reported to date. RHAU/DHX36 recognizes the G4 structures through the RHAU-specific motif (RSM), which is not conserved in DExH1 in terms of primary sequence (Fig. S8). There may be two possible domains in DExH1 to specially recognize rG4s. The first possible domain is the N-terminal RGG domain, which is one of three conserved rG4 recognition domains (3). The second domain is DExH1^45-58^, which is predicted to fold α-helix similar to RSM secondary structure using AlphaFold. In addition, the position of this α-helix in DHxH1 corresponds to the position of RSM in RHAU/DHX36, meaning that their secondary structures are conserved, although the primary sequences are not. The study of the biological functions and molecular mechanisms of the identified helicases modulating rG4s in cells is an exciting research focus.

## Experimental procedures

### Nucleic acid preparations

The sequences of all nucleic acids are listed in Table S1. The RNA substrates used for smFRET were purchased from Dharmacon, and the remaining oligonucleotides were purchased from General Biol. The smFRET oligonucleotides were amino-modified at two specific positions and fluorescently labeled by Cy3 and Cy5 according to previously described protocols (52). Notably, Cy3 and Cy5 were mixed to label substrates at the same time; therefore, there was a 50% chance that one substrate would be labeled with two Cy3s or two Cy5s. This would not have been detectable in smFRET because FRET can only be detected when one Cy3 and one Cy5 are labeled on the same substrate. All of the annealing was performed by incubation at 95 °C for 5 min and then cooled to room temperature for ∼3 h in the corresponding reaction buffers. For simplicity, Kn is used to represent reaction buffer in the text, where n indicates the concentration of KCl; for example, K100 represents 25 mM Tris–HCl at pH 8.0 and 100 mM KCl.

### Protein expression and purification

Full-length genes of *RH3*, *RH53*, *eIF4A1*, *DExH1*, and *DExH15* from *Arabidopsis* complementary DNA were cloned into pET28a and tagged with 6× His-SUMO at their N-terminal. Each expression vector was transformed into *Escherichia coli* Rosetta2 (DE3) and induced with 0.3 mM IPTG at 16 °C for ∼16 h after the *A*_600_ reached ∼0.6 at 37 °C. After the high-pressure homogenization of cells and sequential centrifugation, the expressed protein in the supernatant was captured by nickel-nitrilotriacetate beads. The 6× His-SUMO tags of eIF4A1, DExH1, and DExH15 were removed by Ubiquitin-like protein-specific protease and collected for experimental comparison. In terms of RH3 and RH53, it was found that the removal of their tags decreased their stability in solution; therefore, this tag was retained for them. Finally, gel-filtration chromatography was used to polish the captured protein. All of the purified proteins were frozen in small aliquots in liquid nitrogen and stored at −80°C in a storage buffer (10 mM Tris–HCl, pH 7.9, 200 mM KCl, 1 mM DTT, and 50% glycerol (v/v)). The images of their SDS-PAGE gels are shown in Fig. S6.

### CD spectropolarimetry

The CD analysis was performed on a Chirascan V100 (Applied Photophysics Ltd), equipped with a temperature-controlled cell holder and a temperature probe. The final concentration of substrates was 10 μM in 25 mM Tris–HCl, pH 8.0, and 100 mM KCl. The CD spectra were recorded in a range of 220 to 320 nm with a 0.5 mm path length using a quartz cell. For melting, the temperature was raised 1 °C/min continuously between 20 to 98 °C and spectral data were recorded every 2 min.

### smFRET data acquisition and analysis

The equipment and preparation were the same as described in a previous work (28). An oxygen scavenging system (0.8% D-glucose, 1 mg/ml glucose oxidase [266,600 units/g], 0.4 mg/ml catalase [2000–5000 units/mg], and 4 mM Trolox) was added to the reaction buffer to prevent blinking and bleaching. The reaction buffer for the rG4 dynamics experiments contained 25 mM Tris–HCl, pH 8.0, and a corresponding concentration of KCl. Then, 5 mM MgCl_2_ was added to the reaction buffer for the unfolding study of DExH1. In helicase unfolding experiments, 100 nM DExH1 and 1 mM ATP flowed into the chamber simultaneously. The exposure time of 100 ms was used for all of the measurements at a constant temperature of 22 °C. The FRET efficiency was calculated using *I*_A_/(*I*_D_ + *I*_A_), where *I*_D_ and *I*_A_ represent the intensity of the donor (Cy3) and acceptor (Cy5), respectively. smFRET histograms were generated by selecting 30 to 50 frames of each trace from over about 300 molecules. Data analyses were carried out using scripts written in *R*. Hidden Markov modeling was used to identify folding states (53), and the transition density plots were built using our previous method (28). All of the data fitting was performed by OriginPro 2017.

### Proteomics screen

The plant extraction protocol was conducted as described in a previous work (54), and the pull-down method was conducted as previously described (24, 39). Briefly, 1 mg of streptavidin-coated beads (M-280 Dynabeads, Life Technologies) was washed with 200 μl protein-binding buffer (20 mM Tris–HCl, pH 7.5, 300 mM KCl, 0.1% Tween-20, 2 mM EDTA, and 2‰ RNase inhibitor (v/v) [Promega]) and resuspended to a final concentration of 5 mg/ml. One equal volume of 4 μM biotin-modified RNA was added and incubated for 20 min at 20 °C. The beads were then transferred and washed with pull-down buffer (protein-binding buffer, adding cocktail [Sigma]). One gram samples of 7-day-old *Arabidopsis* were frozen with liquid nitrogen and ground into fine powder. Five milliliters of pull-down buffer was added and centrifuged for 20 min at 4 °C and 16,000*g*, followed by filtration through a 0.20 μm filter. The *Arabidopsis* lysates (5 ml per 1 mg of beads) were incubated with the substrate-conjugated Dynabeads at 4 °C for 4 h, collected by magnetic rack, washed three times using the pull-down buffer, and then eluted with 50 μl SDS-PAGE loading buffer through boiling for 5 min. After two biological repeats for each substrate, the samples were run in an SDS-PAGE gel and subjected to LC-MS/MS analysis (BGI) flowing the published protocol (24).

### MST

The MST assays were carried out using a NanoTemper monolith NT.115 (29). The experimental conditions consisted of 25 mM Tris–HCl, pH 8.0, and 300 mM KCl. The concentration of the fluorescein-labeled substrate was 20 nM. The experiments were performed using 40% LED power and 40% MST, with a laser-on time of 30 s and laser-off time of 5 s, at a constant temperature of 22 °C. The thermophoresis signal was recorded and fitted *via* NTAnalysis from at least two independent experiments.

### Gel shift assays

The rG4 unwinding assays were performed using gel shift according to the procedure described in a published work in three independent repetitions (41). Briefly, different concentrations of purified DExH1 and DExH15 were mixed with 160 nM rG4 substrates in 25 mM Tris–HCl, pH 8.0, 50 mM KCl, 5 mM MgCl_2_, 2 U/μl RNase inhibitor, 10% glycerol, and 1 mM DTT. The mixture was incubated for 10 min at 37 °C. Then, one equal volume of 4 mM ATP was added along with 1.6 μM traps to initiate reactions and incubated for 30 min at 37 °C. Then 5× stop buffer (125 mM EDTA and 50% glycerol (v/v)) and proteinase K (final concentration of 2 mg/ml) were added sequentially. After waiting for 10 min at 37 °C, the reaction products were electrophoresed on a 12% native PAGE. Finally, the gels were imaged using the Cy3 channel on a ChemiDoc MP Visualization System (BioRad) and analyzed in ImageJ (National Institutes of Health).

## Data availability

The mass spectrometry proteomics data have been deposited to the ProteomeXchange Consortium *via* the PRIDE (55) partner repository with the dataset identifier PXD031877.

## Supporting information

This article contains supporting information.

## Conflict of interest

The authors declare that they have no conflicts of interest with the contents of this article.
