# Supplementary material for: Insights into the structural dynamics and helicase-catalyzed unfolding of plant RNA G-quadruplexes
Source: J Biol Chem. 2022 Jun 20;298(8):102165. doi: 10.1016/j.jbc.2022.102165 (PMC9293640; doi:10.1016/j.jbc.2022.102165)
Supplement: Supporting Information [file mmc1.pdf]

Supporting Information

Insights into the structural dynamics and helicase-catalyzed unfolding of plant RNA G-quadruplexes

Liu Wang†, Ya-Peng Xu†, Di Bai, Song-Wang Shan, Jie Xie, Yan Li, Wen-Qiang Wu*

From the State Key Laboratory of Crop Stress Adaptation and Improvement, Academy for Advanced Interdisciplinary Studies, School of Life Sciences, Key Laboratory of Plant Stress Biology, Henan University, Kaifeng 475001, China.

†These authors contributed equally to this work as Joint First Authors.

*To whom correspondence should be addressed.

Tel: +86 371 23881387; Fax: +86 371 23881387; Email: wuwenqiang@henu.edu.cn

Table S1. Sequences of substrates used in the experiments.

Name	For figures	Sequences (5'-3') of substrates for CD
2G (C)	Figure 1	UUUU**CGGCGGCGGCGG**UUUU
2G (AA)	Figure 1	UUUU**GGAAGGAAGGAAGG**UUUU
3G (SMXL)	Figure 1	UUUU**GGGGGUGGGGGGUUAGGGUUAGGG**UUUU
3G (ATR)	Figure 1, S3	UUUU**GGGAGGGAAGGGGAAGGGG**UUUU
3G (ATR)-DNA	Figure S3	TTTT**GGGAGGGAAGGGGAAGGGG**TTTT
		**Sequences (5'-3') of substrates for smFRET**
2G (C)	Figure 2, 3, S1	Biotin-UUUUUUUUUUUUUUUUU (U-NH2) U**CGGCGGCGGCGG**U (U-NH2) UUUUUUUUUUUUU
2G (AA)	Figure 2, 3, S1	Biotin-UUUUUUUUUUUUUUUUU (U-NH2) U**GGAAGGAAGGAAGG**U (U-NH2) UUUUUUUUUUUUU
3G (SMXL)	Figure 2, 3, S1	Biotin-UUUUUUUUUUUUUUUUU (U-NH2) U**GGGGGUGGGGGGUUAGGGUUAGGG**U (U-NH2) UUUUUUUUUUUUU
3G (ATR)	Figure 2, 3, 8, S1	Biotin-UUUUUUUUUUUUUUUUU (U-NH2) U**GGGAGGGAAGGGGAAGGGG**U (U-NH2) UUUUUUUUUUUUU
ATRG3	Figure 4	Biotin-UUUUUUUUUUUUUUUUU (U-NH2) U**GGGAGGGAAGGGG**AAUUUUU (U-NH2) UUUUUUUUUUUUU
3G (ATR)-DNA	Figure S4	Biotin-TTTTTTTTTTTTTTTTT (T-NH2) T**GGGAGGGAAGGGGAAGGGG**T (T-NH2) TTTTTTTTTTTTT
		**Sequences (5'-3') of substrates for proteomics screen**
G4RNA15Ubiotin	Figure 5	GA**GGGAGGGAAGGGGAAGGGG**UAAUUUUUUUUUUUUUUU-biotin
15Ubiotin	Figure 5	UUUUUUUUUUUUUUU-biotin
		**Sequences (5'-3') of substrates for MST**
G4S	Figure 6, S7	**GGGAGGGAAGGGGAAGGGG**UUUUUUUUUUUUUUU-FAM
SG4	Figure 6, S7	FAM-UUUUUUUUUUUUUUU**GGGAGGGAAGGGGAAGGGG**
S	Figure 6, S7	FAM-UUUUUUUUUUUUUUU
DS	Figure 6, S7	GUCUUUACGGUGCUUAAAACAAAACAAAACAAAACAAAA<br>AGCACCGUAAAGAC-FAM
SD	Figure 6, S7	AAAACAAAACAAAACAAAACAAAAUUCGUGGCAUUUCUG<br>FAM-CAGAAAUGCCACGA
		**Sequences (5'-3') of substrates for gel-shift**
3'ss-rG4	Figure 7	Cy3-A**GGGUGGGCCUGGGAGGGG**UGGUGGCCAUUUUUUUUUUU
3'ss-rG4 trap	Figure 7	UGCUGGCCCGUUCGCCCCUCCCGGG
5'ss-rG4	Figure 7	UUUUUUUUUUUACCGGUGGU**GGGGAGGGUCCGGGUGGG**A-Cy3
5'ss-rG4 trap	Figure 7	GGGCCCUCCCCGCUUGCCCGGUCGU

Color: blue, FAM; Green, Cy3. **Bold**: **G4/G3 sequences**. Underline: dsRNA forming sequences.

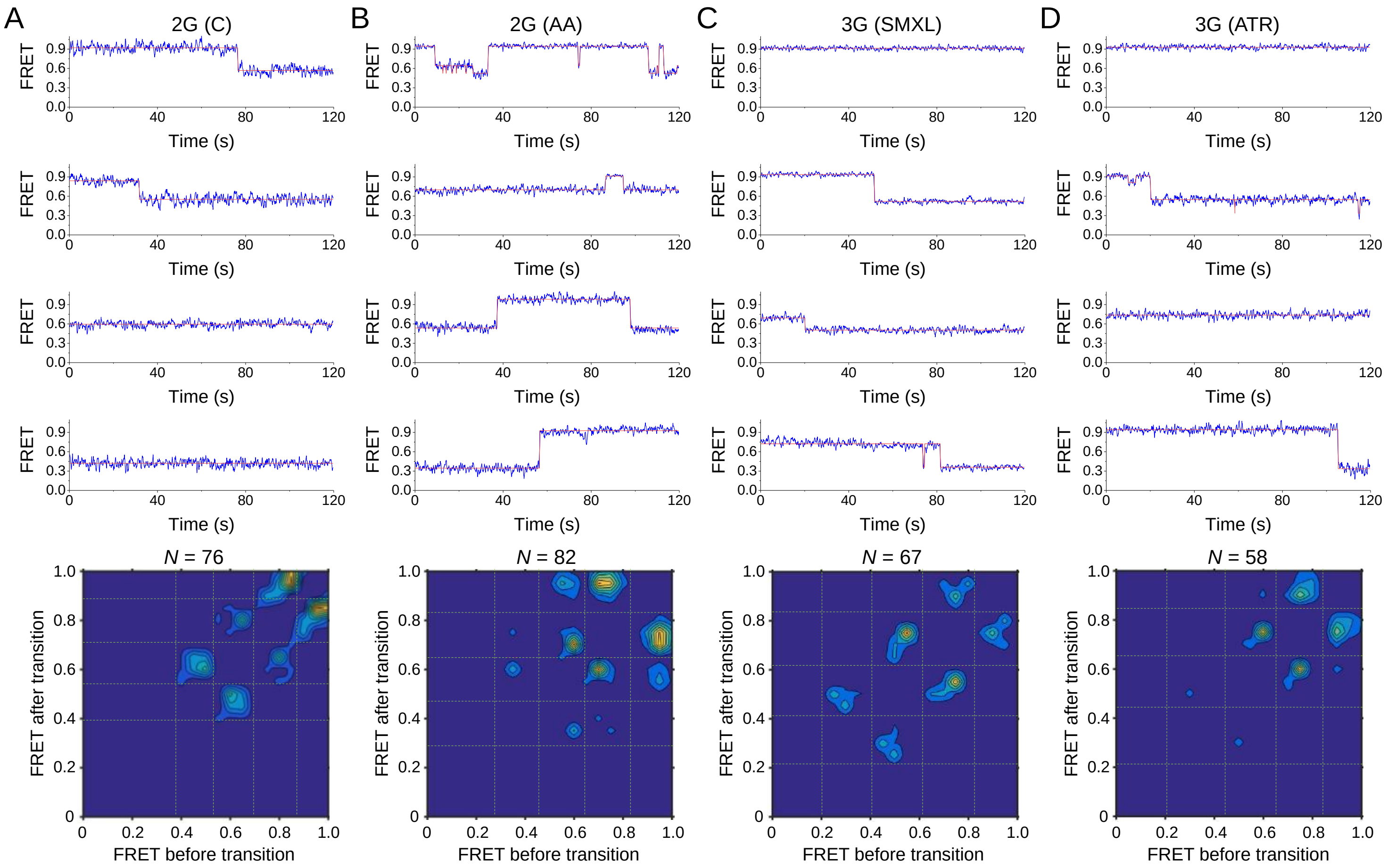

Figure S1. The representative traces of 2G (C), 2G (AA), 3G (SMXL), and 3G (ATR) in 25 mM Tris-HCl, pH 8.0, and 100 mM KCl (upper panel). Dynamic states are determined by hidden Markov modeling (red line), and the transition density plots of all four rG4s are built showing four states (lower panel). *N* represents the number of traces used for analysis.

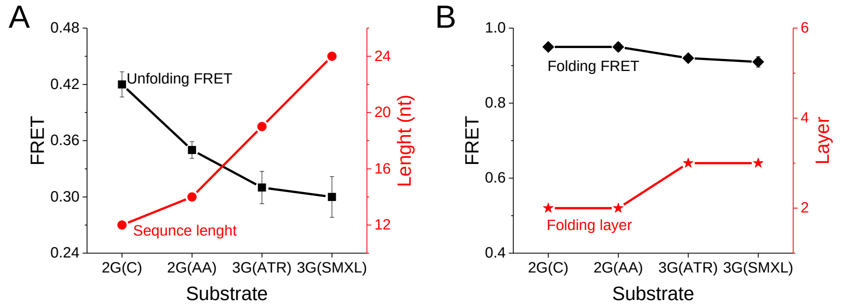

Figure S2. FRET change of unfolded states with the length of single strands (A) and folded states with the number of tetrad layers (B).

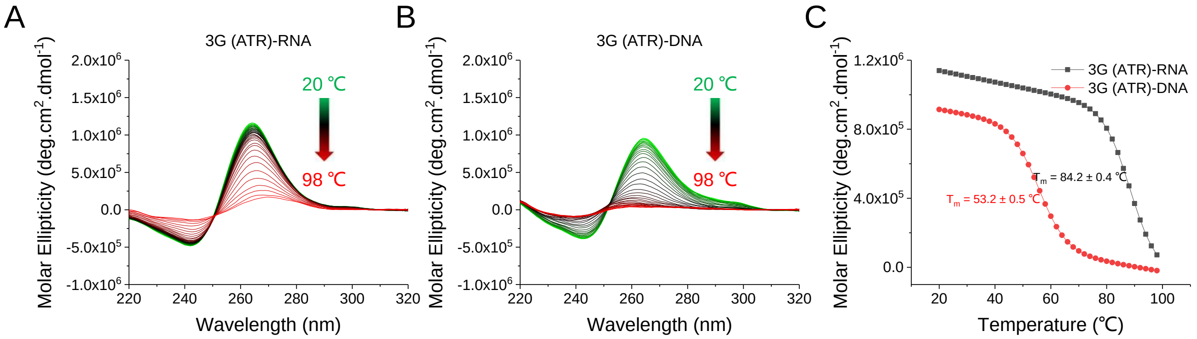

Figure S3. T_m value measurements of 3G (ATR)-RNA and 3G (ATR)-DNA. (A, B) The melting CD spectra of 3G (ATR)-RNA (A) and 3G (ATR)-DNA (B) in 25 mM Tris-HCl, pH 8.0, and 100 mM KCl. (C) T_m fitting using the 264 nm CD values from (A) and (B).

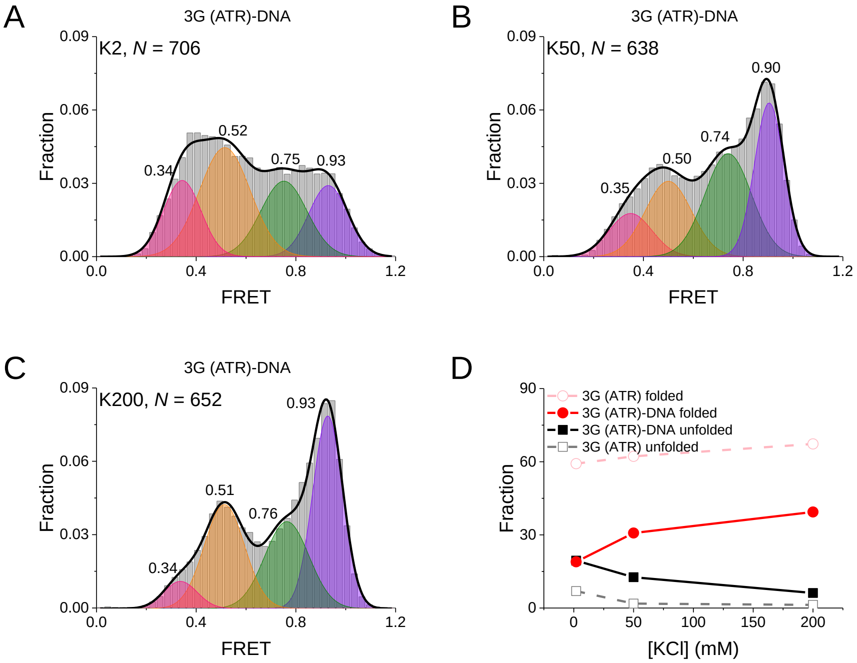

Figure S4. Histogram fitting of 3G (ATR)-DNA using multipeak Gaussian distributions showing four peaks (A–C), and the comparison of well-folded and unfolded fractions of 3G (ATR)-DNA with 3G (ATR)-RNA (D). The data of 3G (ATR)-RNA are obtained from Figure 3. *N* represents the number of traces used for analysis.

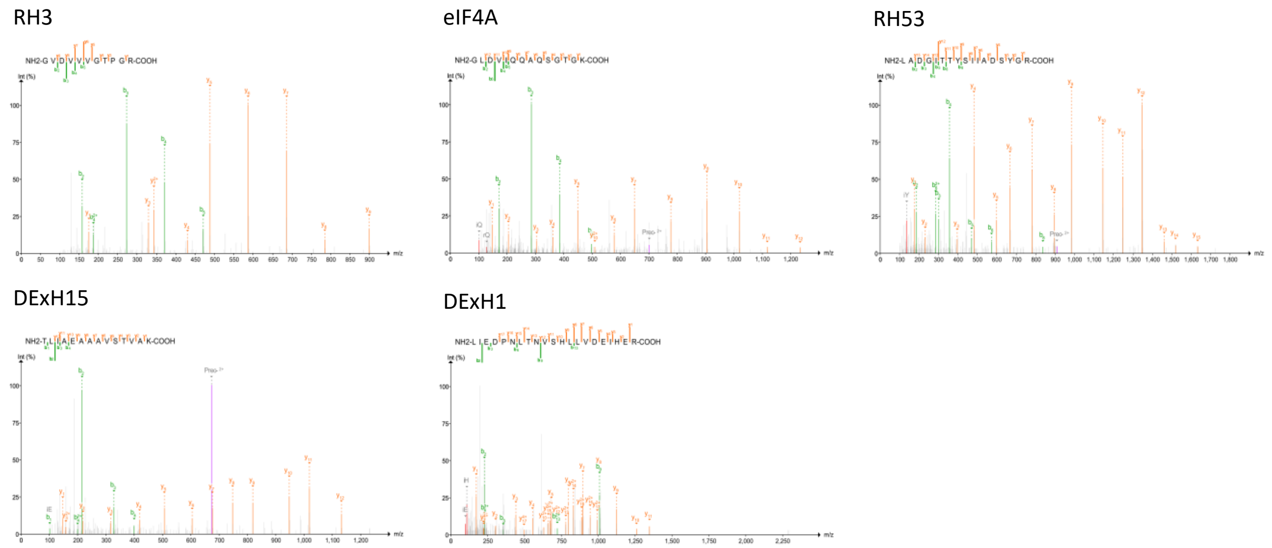

Figure S5. The mass spectrometric spectrum of representative peptides.

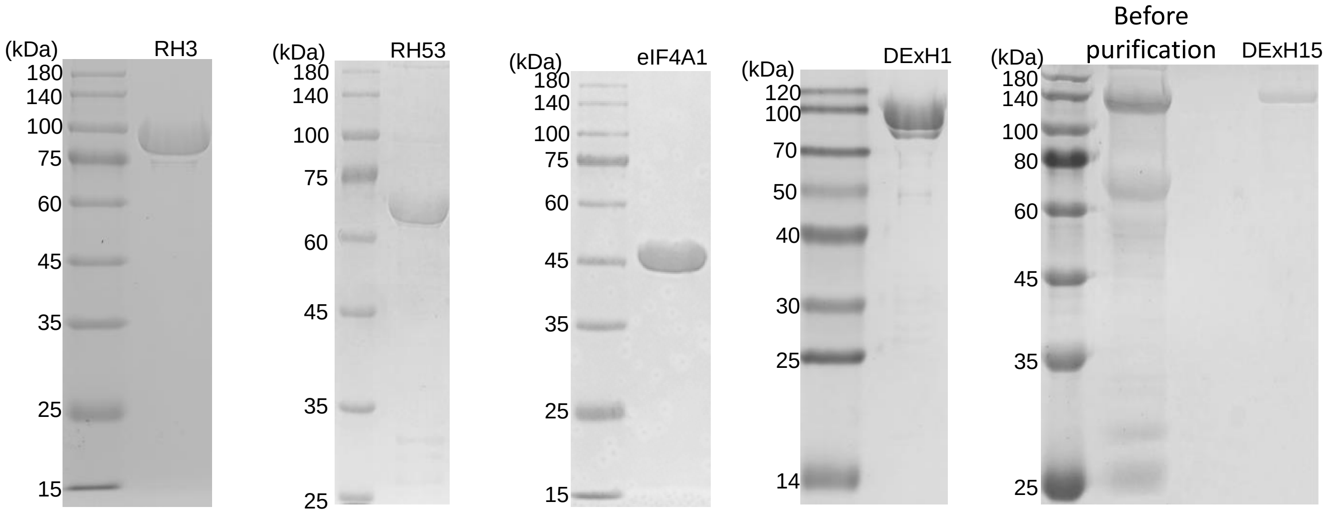

Figure S6. SDS-PAGE images of the five purified helicases.

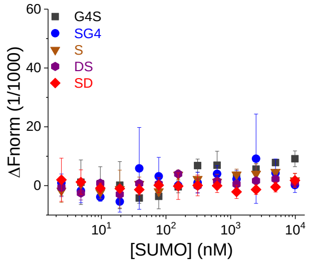

Figure S7. The MST signals of SUMO binding different substrates.

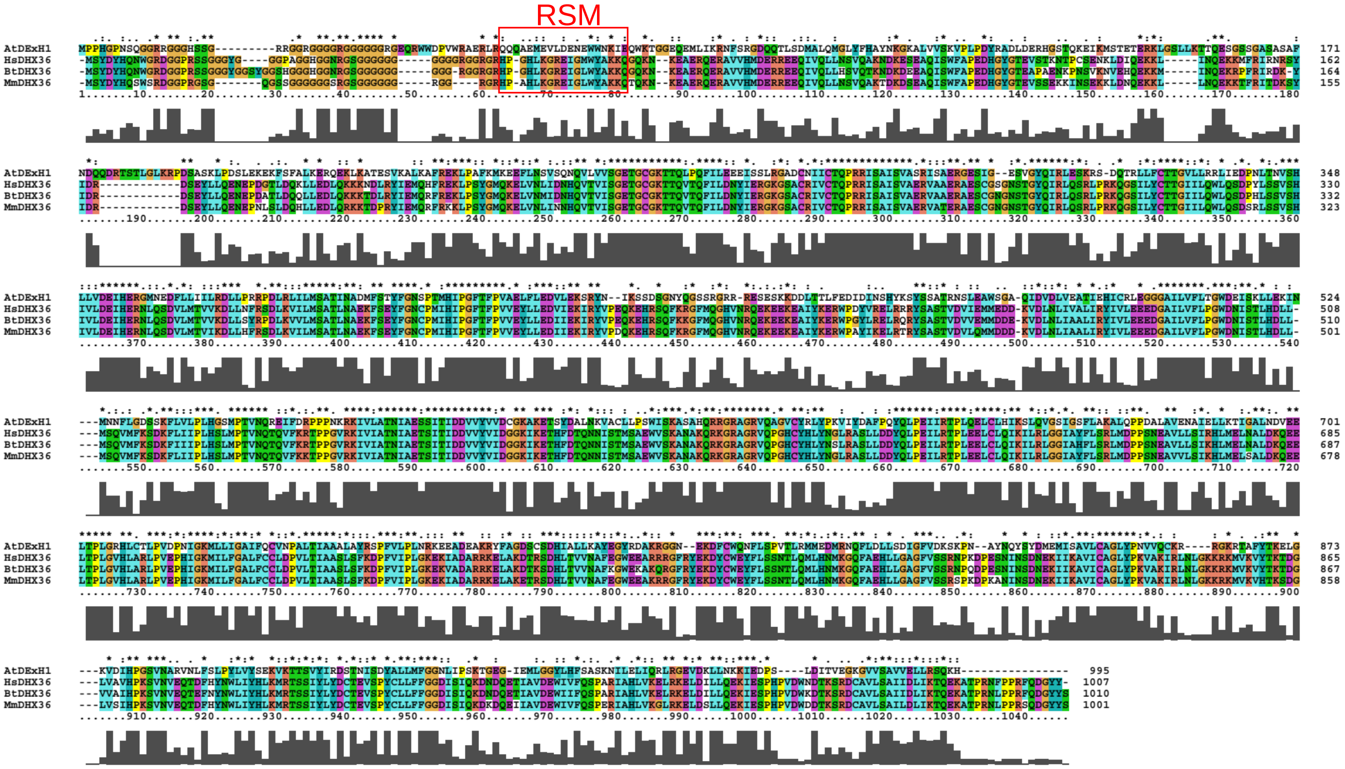

Figure S8. Sequence comparison of DExH1 from *Arabidopsis thaliana* (At), *Homo sapiens* (Hs), *Bos taurus* (Bt), and *Drosophila melanogaster* (Dm). RHAU-specific motif (RSM) is not conserved in terms of primary sequence.

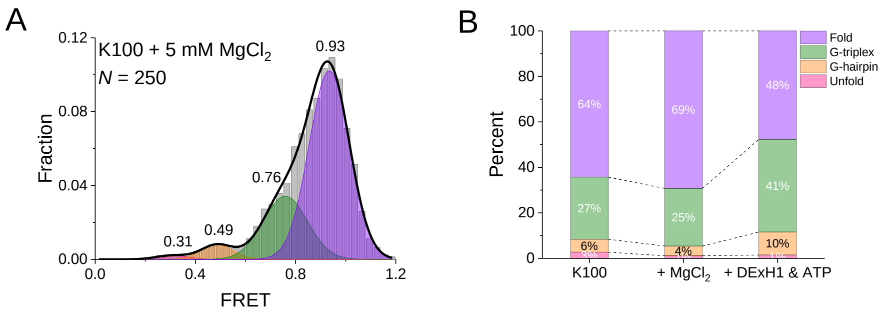

Figure S9. (A) FRET distributions of 3G (ATR) in the presence of 100 mM KCl and 5 mM $MgCl_2$, using multipeak Gaussian distributions showing four peaks. (B) The fractions of different folding states in the presence of K100 (100 mM KCl), +$MgCl_2$ (100 mM KCl and 5 mM $MgCl_2$), and +DExH1 and ATP (100 mM KCl, 5 mM $MgCl_2$, 100 nM DExH1, and 1mM ATP). *N* represents the number of traces used for analysis.
